# Xuebijing injection mitigates instant blood−mediated inflammatory reaction and enhances intrahepatic islet transplantation via target NF−κB pathway

**DOI:** 10.3389/fimmu.2025.1671966

**Published:** 2025-12-23

**Authors:** Yixiang Zhan, Yingbo Wang, Boya Zhang, Yijun Zhang, Rui Liang, Jiuxia Yang, Tengli Liu, Xiaoyan Hu, Tianyi You, Na Liu, Yuqi Chen, Qing Liu, Tingsheng Jiang, Zhaoce Liu, Xiangheng Cai, Runnan Yang, Yingyi Qi, Peng Sun, Jiaqi Zou, Xuejie Ding, Zhuzeng Yin, Shusen Wang

**Affiliations:** 1 Research Institute of Transplant Medicine, Organ Transplant Center, National Health Commission (NHC) Key Laboratory for Critical Care Medicine, Tianjin First Central Hospital, School of Medicine, Nankai University, Tianjin, China; 2 The First Central Clinical College, Tianjin Medical University, Tianjin, China; 3 Faculty of Hepato-Pancreato-Biliary Surgery, The First Medical Center of Chinese People’s Liberation Army (PLA) General Hospital, Beijing, China

**Keywords:** islet transplantation, IBMIR, Xuebijing injection, NF-κB pathway, diabetes

## Abstract

**Introduction:**

Instant blood-mediated inflammatory reaction (IBMIR) is a major obstacle in clinical islet transplantation, leading to islet apoptosis and dysfunction due to inflammatory reaction. Xuebijing (XBJ), a traditional Chinese medicine, has been extensively used in the treatment of systemic inflammatory conditions and achieved remarkable effect. Giving these properties, XBJ holds promise in improving the outcomes of intrahepatic islet transplantation through inhibiting IBMIR.

**Methods:**

The xenogeneic islet transplantation model was employed to evaluate the inhibitory effects of XBJ on IBMIR, while the syngeneic transplantation model was used to confirm that XBJ improves the long-term outcomes of intrahepatic islet transplantation through IBMIR suppression. In addition, studies were conducted under inflammatory conditions to demonstrate the protective effects of XBJ on islets in vitro, specifically its ability to preserve islet viability and function in an inflammatory environment.

**Results:**

*In vivo* IBMIR model, XBJ significantly inhibited leukocyte infiltration, leading to reduced islet damage. *In vitro*, XBJ provided direct protection to islets in inflammatory stimulation, preventing apoptosis and preserving islet function. These protective effects were further demonstrated in the syngeneic islet transplantation model, where XBJ markedly improved the outcomes of intrahepatic islet transplantation.

**Discussion:**

This study provides the evidence that XBJ improves islet transplantation outcomes through dual mechanisms targeting the IBMIR. As an already approved drug, XBJ presents a promising and readily translatable adjunctive therapy for clinical intrahepatic islet transplantation.

## Introduction

Islet transplantation has emerged as a promising therapeutic option for type 1 diabetes (T1D), offering the potential for insulin independence and improved glycemic control (1, 2). Despite these promising outcomes, the success of intrahepatic islet transplantation is often compromised by a complex and detrimental immune response known as the instant blood-mediated inflammatory reaction (IBMIR), a severe immune response that occurs immediately post-transplantation (3). IBMIR leads to more than 50% islet cells loss through apoptosis and necrosis immediately post-transplantation (4, 5), compromising graft function and long-term transplant success. Early innate immune responses diminish the overall efficacy of the procedure. Despite advances in immunosuppressive therapies and anti-inflammatory strategies, IBMIR remains a major challenge to improve islet grafts survival.

The pathogenesis of IBMIR involves a vicious cycle between innate immunity and coagulation activation (6). Upon exposure to blood, the islet grafts trigger inflammatory response immediately, characterized by the release of pro-inflammatory cytokines and the activation of immune cells such as neutrophils and macrophages. This innate immune activation exerts direct cytotoxic effects on islet cells and also contributes to the initiation of the adaptive immune response (7). Concurrently, the interplay between inflammation and coagulation creates a cascade amplification effect. Critically, this synergistic amplification leads to mass β-cell apoptosis within hours after transplantation, which represents the hallmark of IBMIR (8). The extensive loss of islets severely compromises engraftment efficiency and glycemic control. This synergistic amplification is central to the destructive nature of IBMIR and underscores the urgent need for novel interventions that can interrupt this cycle and improve islet grafts outcomes.

Given the multifactorial nature of IBMIR and the concurrent need to directly protect islet cells from inflammatory injury, a dual-action therapeutic agent would offer superior benefits. Xuebijing (XBJ) is an injectable formulation approved by China’s National Medical Products Administration for intravenous use in acute conditions, which is a standardized Chinese herbal extract with demonstrated efficacy in sepsis, multiple organ dysfunction syndrome and COVID-19 (9–11). Clinically, XBJ had demonstrated remarkable clinical efficacy in treating a variety of acute inflammatory injuries, particularly in the context of severe infections like sepsis (12, 13). Crucially, a double-blind, placebo-controlled, multicenter study had been proved the effect of XBJ in reducing mortality among patients with sepsis (14). Notably, XBJ suppresses the excessive and uncontrolled release of pro-inflammatory cytokines, which is the key feature of severe acute inflammatory reaction (15, 16). Since IBMIR is characterized by early cytokine aggregation, which not only mediates inflammation but also leads to substantial graft loss (17–19). By modulating these critical pathways, XBJ has the potential to reduce islet apoptosis, improve graft survival and enhance the overall success of islet transplantation.

Based on these evidences, we hypothesized that XBJ could provide a novel therapeutic strategy to improve graft survival and enhance outcomes in intrahepatic islet transplantation. To validate this hypothesis, we investigated the effects of XBJ on IBMIR using both *in vitro* and *in vivo* models of islet transplantation and to assess its potential to enhance islet grafts survival and function. To our knowledge, this is the first study to investigate the application of a traditional herbal formulation in intrahepatic islet transplantation. We propose that the pleiotropic effects of XBJ on NF-κB signaling, and apoptosis inhibition could address the multifactorial mechanisms underlying IBMIR. By integrating mechanistic investigations with preclinical transplantation models, we aim to provide novel insights into the protective effects of XBJ and its potential as an adjunct therapy for enhancing the efficacy of islet transplantation.

## Materials and methods

### Animals

Mouse studies were approved by Nankai University Institutional Animal Care and Utilization Committee (approval NO. 2023-SYDWLL-000621). Male C57BL/6J mice (6–8 weeks old) and Sprague-Dawley rats (6–8 weeks old) were purchased from Beijing Huafukang Biosciences (Beijing, China).

### Islet isolation

Mouse and rat islets were isolated using a standardized collagenase digestion protocol. Briefly, C57BL/6J mice were sacrificed by cervical dislocation, and Sprague-Dawley rats were euthanized by CO_2_ asphyxiation. And the pancreas were perfused with an enzyme solution containing 0.5 mg/mL collagenase P (11213865001, Roche, Basel, Switzerland), 1% HEPES (15630130, Gibco, Carlsbad, United States), 1.27 mM CaCl_2_ in D-hanks solution (H1045, Beijing Solarbio Science & Technology Co., Ltd, Beijing, China). The pancreas were gently removed and the digestion was carried out at 37°C for 11 min, followed by purification by density gradient (Histopaque 1077, Sigma-Aldrich, St Louis, MO, USA).

### Islet viability and apoptosis

The viability assessment of mouse islets were carried out using a dual fluorescent staining method with fluorescein diacetate (FDA, 0.46 μM) and propidium iodide (PI, 14.34 μM), both purchased from Invitrogen (Thermo Fisher Scientific, MA, USA). Islets were exposed to FDA and PI in PBS for a duration of 2 minutes. Subsequently, fluorescence microscopy was employed to capture green (FDA) and red (PI) signals, representing live and dead cells, respectively (ECLIPSE Ni-U microscope, Nikon, Tokyo, Japan). The viability (%) of each islet= [1– PI positive area/(FDA positive area + PI positive area)] ×100%.

The whole islets underwent staining for TUNEL assay performed with the DeadEnd Fluorometric TUNEL system (G3250, Promega, Wisconsin, USA). For each islet, optical section images starting at the peripheral cell layers were acquired using a Nikon A1+ confocal microscope.

### Glucose-stimulated insulin secretion

GSIS assay were conducted as we previously described (20). Briefly, The Krebs-Ringer bicarbonate (KRB) was prepared as following: 125 mM NaCl, 5.9 mM KCl, 2.56 mM CaCl_2_·2H_2_O, 1.2 mM MgCl_2_·6H_2_O, 25 mM HEPES, 1 mg/ml BSA and 1 mM glutamine (pH 7.2–7.4).12 mice islets were pre-incubated in 1 mL 1.67 mM low-glucose KRB buffer supplemented with 0.5% bovine serum albumin for 1h, then the medium was removed. The islets were further incubated in 1 mL 1.67 mM low-glucose and 16.7 mM high-glucose KRB solution for 1 hour sequentially, then the supernatant was collected. Insulin concentration of the supernatant was measured by enzyme-linked immunosorbent assay (ELISA, MS200, EZassay, Shenzhen, China). The glucose-stimulated index (GSI) = insulin content in the 16.7 mM glucose media/insulin content in the 1.67 mM glucose media.

### Islet transplantation

Streptozotocin (STZ, 200 mg/kg, Sigma-Aldrich, St Louis, MO, USA) was injected intraperitoneally into C57BL/6J to induce diabetes. The mice were considered diabetic when non-fasting blood glucose levels exceeded 20.0 mmol/L on 2 consecutive days. For syngeneic and xenograft islet transplantation model, 200 C57BL/6J mice’s islets or 500 Sprague-Dawley rats’ islets were transplanted into the liver of diabetic C57BL/6J recipients via the portal vein respectively.

In xenograft islet transplantation model, liver tissue was collected 6 hours post transplantation to confirm the leukocytic infiltration and islets destruction by pathological analysis. In syngeneic islet transplantation model, non-fasting blood glucose levels were monitored for 45 days after transplantation. Non-fasting blood glucose levels lower than 11.1 mmol/L for two consecutive days were seen as normoglycemia.

### Xuebijing injection

XueBiJing injection was obtained from Tianjin Chasesun Pharmaceuticals (Tianjin, China). C57BL/6 mice were injected with Xuebijing (16 μL/g) via the tail vein one hour before transplantation, followed by a second dose (8 μL/g) administered via the tail vein at 6 hours post-transplantation. Additionally, they received 8μL/g Xuebijing injected twice for three consecutive days post-transplantation. Control mice received saline at the same schedule.

### Intraperitoneal glucose tolerance test

Intraperitoneal glucose tolerance tests (IPGTT) were performed at 30 days after transplantation. The recipient mice were fasted overnight, then received an intraperitoneal glucose dose of 2 g/kg. Blood glucose levels were measured at 0, 15, 30, 60, 90, and 120 min after injection.

### Tissue immunohistochemistry staining

Paraformaldehyde-fixed liver tissues were embedded in paraffin and cut into 5 μm thick serial sections. Sections were stained with hematoxylin and eosin (G1076, Servicebio, Wuhan, China) and examined for islet grafts using ECLIPSE Ni-U microscope (Nikon, Tokyo, Japan).

For immunohistochemistry staining, tissue sections were incubated overnight with anti-CD45 antibody (GB113886, Servicebio, Wuhan, China), along with secondary HRP conjugated goat anti-rabbit IgG (GB23303, Servicebio, Wuhan, China).

For immunofluorescence staining, primary antibodies used anti-insulin (ab63820, Abcam, Cambridge, UK) and anti-glucagon antibody (ab10988, Abcam, Cambridge, UK) incubated overnight. Secondary antibodies used TRITC (111025003, Jackson, Cambridge, UK) and FITC (112095003, Jackson, Cambridge, UK) conjugated with insulin and glucagon respectively. DAPI was used for nuclear counterstaining. Pannoramic MIDI and Pannoramic Viewer (3DHistech, Budapest, Hungary) were used to scan stained slides and capture images. For H&E staining, liver sections were stained with hematoxylin and eosin (G1076, Servicebio, Wuhan, China) and examined for islet grafts using ECLIPSE Ni-U microscope (Nikon, Tokyo, Japan). Thrombus deposition around islets was semi quantitatively analyzed using a scoring scheme. The thrombus deposition rate was evaluated using the following formula: thrombus deposition rate = thrombus area/(thrombus area + islet area)] ×100%. (score 0: rate <10%; score 1: 10-20%; score 2: 20-50%; score 3: rate >50%).

Tissue slides also underwent staining for TUNEL assay performed with the DeadEnd Fluorometric TUNEL system (G3250, Promega, Wisconsin, USA).

### Cell viability

The NIT-1 cells were seeded at a density of 3 × 10^4^ per well in 96-well plates. After overnight incubation, the supernatant was discarded and medium containing the inflammatory cytokines and appropriate concentration of XBJ was added and incubated for 24 hours. Cell viability was analyzed using the CCK8 kit (CCK8, Dojindo, CK04) according to the manufacturer’s instructions. The optical density (OD) value was measured using a microplate reader (EnSpire, PerkinElmer, Massachusetts, USA) at a wavelength of 450 nm.

### Cytokines stimulation

Using a variety of inflammatory factors *in vitro* to mimic the inflammatory damage after transplantation *in vivo*, including: interleukin (IL)-1β (10 ng/ml, 211-11B, Peprotech, Rocky Hill, NJ, USA), TNF-α (25 ng/ml, 315-01A, Peprotech, Rocky Hill, NJ, USA), INF- γ (100 ng/ml, 315-05, Peprotech, Rocky Hill, NJ, USA). Mouse islets or NIT-1 cells were cultured in 6-well plates, and the corresponding concentration of Xuebijing (Tianjin Chase Sun Pharmaceutical Co., Ltd., Tianjin, China) was added, and the experiment was carried out after treatment for 24 h.

### Western blot

After SDS-PAGE for PVDF membrane blotting, the membranes were blocked with 5% skim milk and incubated with primary antibodies Cleaved-Caspase-3 (9661S, Cell signaling technology, Danvers, MA, USA), Caspase-3 (9662S,Cell signaling technology, Danvers, MA, USA), Cleaved-PARP (ab32064, Abcam, Cambridge, MA, USA), Phospho-NF-κB p65 (Ser536) (3033S, Cell signaling technology, Danvers, MA, USA), NF-κB p65 (4764S, Cell signaling technology, Danvers, MA, USA), β-actin (3700S, Cell signaling technology, Danvers, MA, USA). In P65-related assays, we first detect the molecular expression level of P-P65, then perform membrane stripping, followed by detection of P65. Detection of immunoreactive protein bands was achieved via enhanced chemiluminescence, utilizing secondary antibodies conjugated to horseradish peroxidase (HRP). Signal intensity from the resulting bands was evaluated through densitometric analysis and subsequently quantified using ImageJ software (NIH, Bethesda, Maryland, USA).

### RNA-sequencing

The transcriptome analysis was conducted by LC Bio Technology CO., Ltd (P. R. China). Following 24 hours *in vitro* under inflammatory cytokines stimulation with or without XBJ, RNA libraries were prepared from RNA lysate isolated from 500 mouse islets each sample. In brief, RNA was isolated using TRIzol reagent and RNA quality was evaluated with NanoDrop ND-1000 (NanoDrop, Wilmington, DE, USA). The RNA integrity was assessed by Bioanalyzer 2100 (Agilent, CA, USA) with RIN number>7.0, and confirmed by electrophoresis with denaturing agarose gel. With high-quality RNA, cDNA library was created and then the 2x150bp paired-end sequencing was performed on an Illumina Novaseq 6000. Bioinformatic analysis was performed using the OmicStudio tools. Differentially expressed genes (DEGs) were identified using DESeq, with criteria set to a false discovery rate (FDR) of <0.05 and a log2 |fold-change|≥2. The plots were drawn based on the R version 4.1.3 on the OmicStudio platform. DEGs were subjected to Kyoto Encyclopedia of Genes and Genomes (KEGG) pathway enrichment analysis and Gene Ontology (GO) enrichment analysis. These analyses were performed using the OmicStudio tools (https://www.omicstudio.cn/tool).

### Real-time polymerase chain reaction

Total RNA was extracted from NIT-1 cells, islets or livers samples using Trizol (Invitrogen, Carlsbad, CA, USA) according to the manufacturer’s instructions. Total RNA was reverse transcribed to cDNA using a reverse transcriptase reaction kit (Takara, Kohoku-cho, Kusatsu, Japan). qRT-PCR was performed using FastStart Essential DNA Green Master on a LightCycler96 machine (Roche, Basel, Switzerland). The relative expression of mRNA to internal control (β-actin RNA) was calculated using the 2^-ΔΔCt^ method. The primers used to assess the expression of mRNA are shown in **Supplementary Table 1**.

### Molecular docking

The 2D molecular structures of phytochemicals were downloaded from the PubChem database (https://pubchem.ncbi.nlm.nih.gov), and converted into 3D patterns and optimized by Chem3D software. Additionally, the protein structures of ligand-receptors were derived from the Protein Data Bank platform (PDB, https://www.rcsb.org), and then subsequently edited by AutoDock software. Vina, a docking tool of AutoDock software, was used to perform the docking of these small molecules and protein structures. The criteria for qualified docking of each couple is that at least one amino acid residue can be attached to the small molecule within the active region of the protein structure, while the absolute value of the docking score is greater than 5 kcal/mol. These data are eventually imported into PyMol software for visualization.

### Statistical analysis

Quantitative results are presented as the mean ± standard error of the mean (SEM). Statistical analyses were performed using either Student’s t-test or one-way/two-way ANOVA. The rates of normoglycemia following islet transplantation were evaluated by applying the log-rank (Mantel–Cox) test alongside the Gehan–Breslow–Wilcoxon method. A p-value below 0.05 was interpreted as indicating statistical significance.

## Results

### XBJ inhibits IBMIR through suppressing inflammation and thrombosis

**Figure 1:**
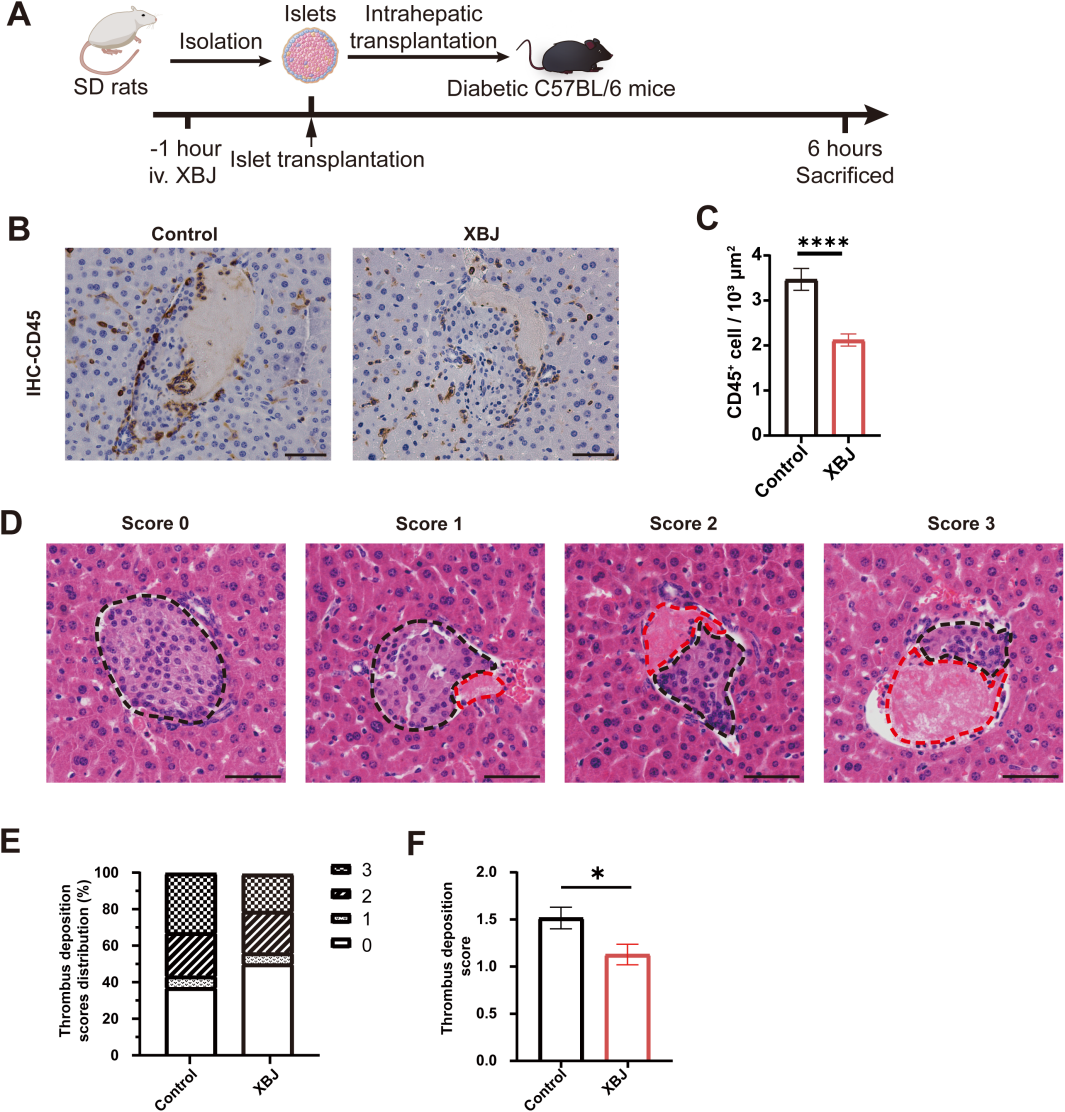
XBJ attenuates IBMIR in intrahepatic islet transplantation model. **(A)** Schematic representation of the xenogeneic islet transplantation timeline. **(B)** Representative immunohistochemical staining images of CD45 around islets graft from both groups. **(C)** Quantification of CD45+ cells infiltration around islets for each group. **(D)** Representative histological images of liver sections showing islet grafts with different levels of thrombus deposition. **(E)** Distribution of thrombus deposition scores in control and XBJ treatment group. **(F)** Quantification of thrombus deposition scores for each group. Data are presented as the mean ± SEM. Scale bar, 50μm. *p <0.05, ****p <0.0001.

To investigate the inhibitory effect of XBJ on IBMIR, we analyzed the coagulation and immune response in intrahepatic islet transplantation model (**Figure 1A**). Liver tissues were harvested 6 hours post-transplantation and subjected to histological analysis. The inflammatory response activated immediately after the islets meet blood is one of the major characteristic features of IBMIR. We stained the adjacent sections to the insulin-positive sections using CD45, a leukocyte common antigen and marker for inflammation, to evaluate immune cell infiltration in the transplanted islets (**Figures 1B, C**). The results demonstrated that leukocyte infiltration was markedly reduced around islets in the XBJ treatment group compared to the control group (**Figure 1C**).

The overactivation of the innate immune system and the coagulation cascade is the indispensable characteristic manifestations of IBMIR. Using a semi-quantitative scoring scheme to evaluate the status of thrombus deposition around islets (**Figure 1D**) (21), we observed that with XBJ treatment reduced peri-islet thrombus deposition from an average score of 1.49 to 1.23 (**Figures 1E, F**; **Supplementary Figure S1**). Notably, 46.19% of islets in the XBJ treatment group had a thrombus deposition of 0 score compared to only 34.95% in the control group.

### XBJ alleviates IBMIR-induced early islet loss and apoptosis

**Figure 2:**
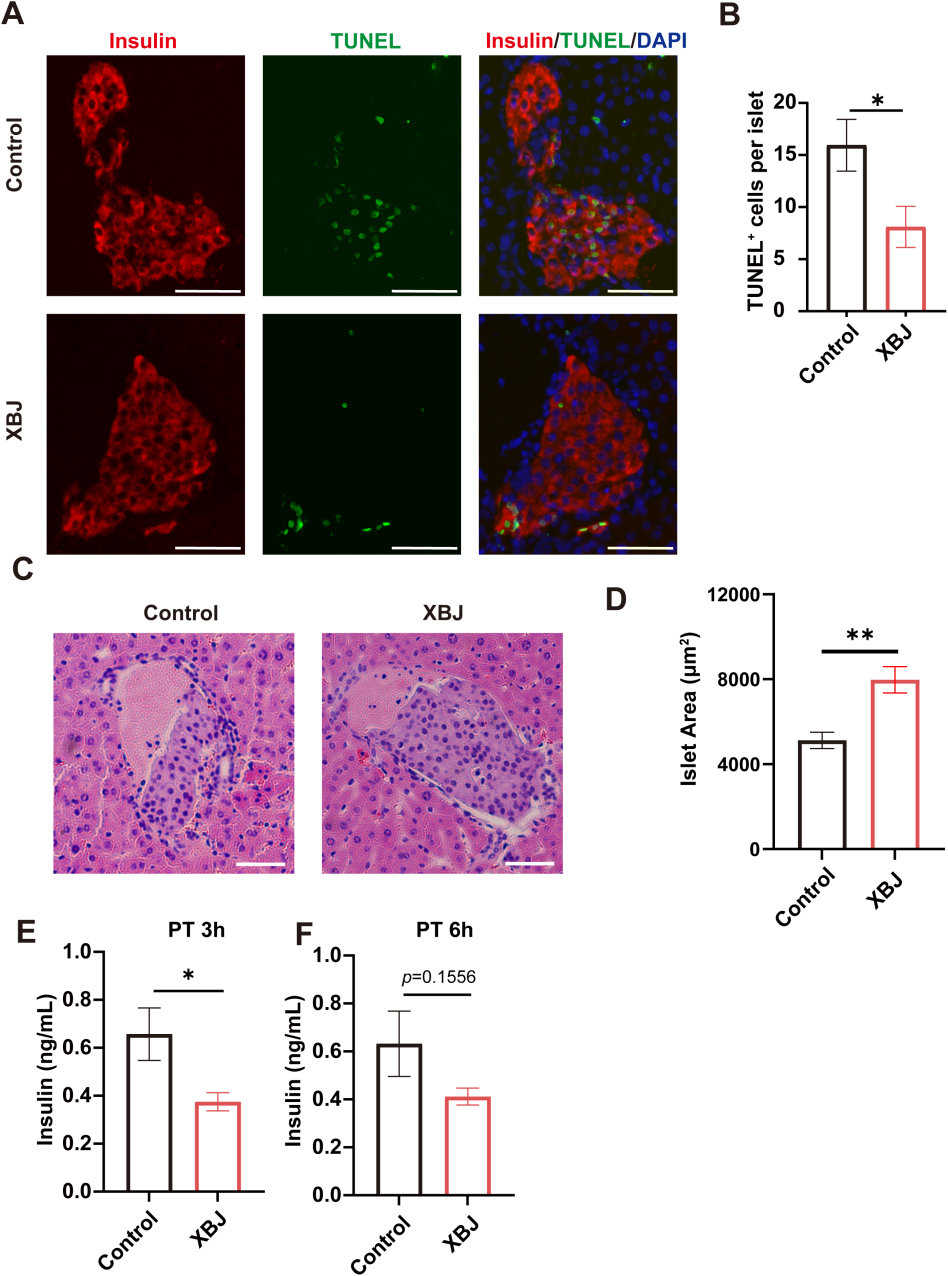
XBJ attenuates islet apoptosis and preserves islet grafts mass during the early phase after intrahepatic transplantation. **(A)** Representative images of TUNEL staining of islet grafts at 6 hours post-transplantation. **(B)** Quantification of TUNEL-positive cells. **(C)** Representative images of islet grafts at 6 hours post-transplantation. **(D)** Quantitative analysis of per islet area. **(E, F)** Serum insulin concentrations at 3 h **(E)** and 6 h **(F)** post-transplantation. Data are presented as mean ± SEM. Scale bar, 50μm. *p <0.05, **p<0.01.

The effect of XBJ on IBMIR-induced apoptosis was assessed by TUNEL staining (**Figure 2A**). We demonstrated significantly lower TUNEL-positive graft islet cells in XBJ treatment group compared with control group (**Figure 2B**). This indicates that XBJ significantly alleviates islet apoptosis induced by IBMIR. Histological staining confirmed a significantly larger islet area in the XBJ-treated group compared to controls. (**Figure 2C**). Islet grafts lost manifested as the islet area had sharply decreased in control group. Conversely, there was a significant higher per islet area in XBJ treatment group (**Figure 2D**), suggesting better preservation of islet mass compared with control group.

The excessive and disorderly release of insulin in the early stage after transplantation may be related to the destruction of the islets (22). Serum insulin levels were assessed in recipients at 3h and 6h post transplantation (**Figures 2E, F**). Notably, the XBJ-treated group exhibited a significantly lower insulin concentration compared to the control group in 3h post transplantation. Collectively, these findings indicate that XBJ effectively alleviates islet apoptosis and preserved islet grafts mass.

### XBJ directly protects NIT-1 and islet cells under cytokine-induced stress *in vitro*

**Figure 3:**
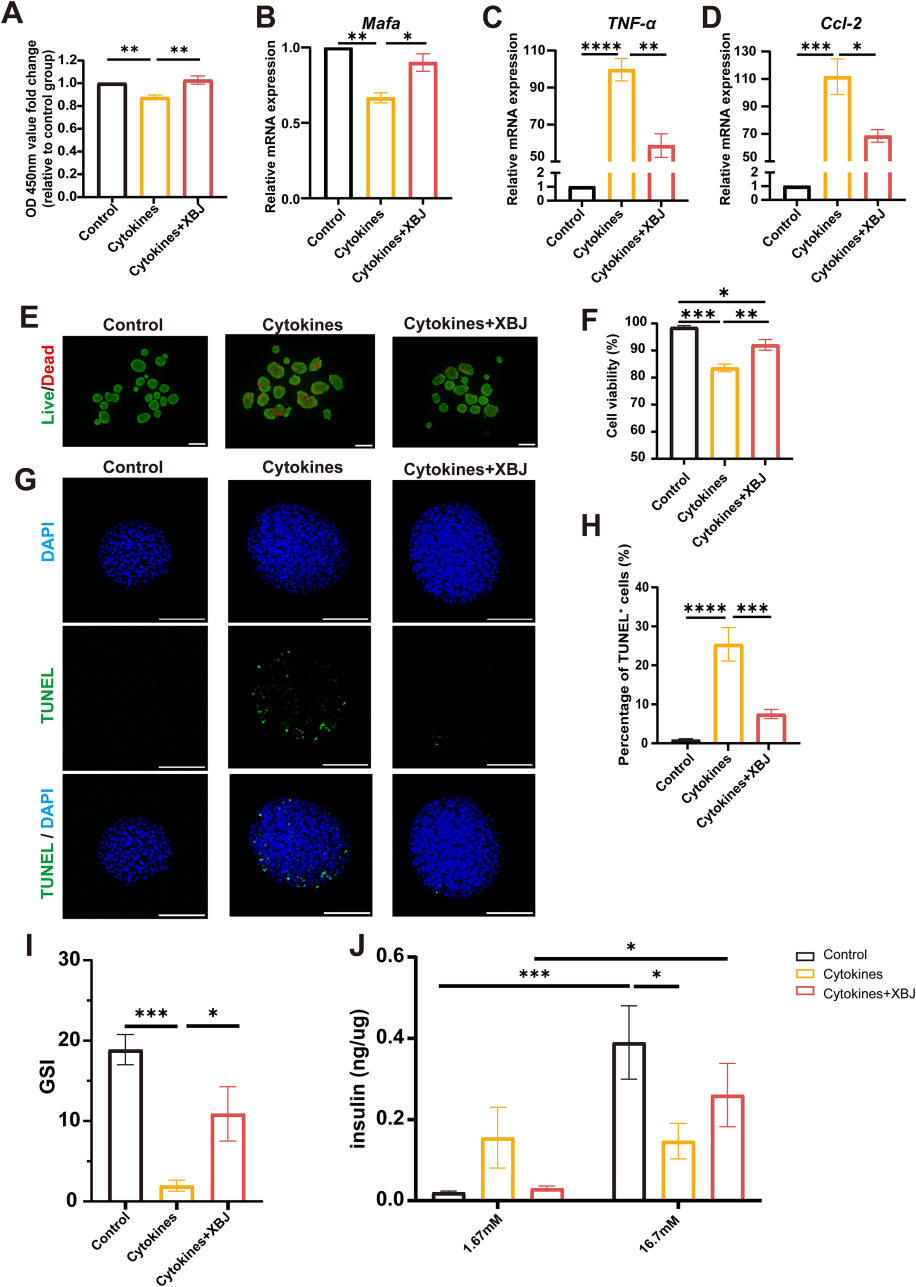
XBJ improves β cells viability under cytokines stimulation. **(A)** The NIT-1 cells viability under inflammatory cytokines stimulation with or without XBJ (n=5). **(B-D)** qRT-PCR analysis of NIT-1 cells under inflammatory cytokines stimulation with or without XBJ (n=3). **(E)** Representative images of FDA/PI staining of islets showing the viability of mouse islets in inflammatory stimulation with or without XBJ. **(F)** Quantitative evaluation of islet viability (n=4). **(G)** Representative immunofluorescence images of TUNEL (green) and DAPI (blue) of mouse islets in inflammatory stimulation with or without XBJ. **(H)** Quantitative evaluation of islet cells apoptosis (n≥12, from 3 independent trials). **(I)** GSI of mouse islet in inflammatory stimulation with or without XBJ, (n= 5). **(J)** Insulin secretion levels after low glucose and high glucose stimulation during glucose-stimulated insulin release assay, (n= 5). Data are presented as the mean ± SEM. Scale bar, 100μm. *p<0.05, **p<0.01, ***p<0.001, ****p<0.0001.

To mimic the inflammatory microenvironment encountered by transplanted islets, we used *in vitro* multiple cytokines stimulation models. Under cytokines stimulation, XBJ effectively alleviated the damage caused by cytokines stimulation in NIT-1 cells through CCK8 measurement (**Figure 3A**). Furthermore, XBJ could upregulate β-cell function-related genes and downregulate pro-inflammatory cytokine gene expression (**Figures 3B–D**).

This protective effect was further confirmed in primary mouse islets. Following a 24-hour cytokine stimulation, FDA/PI staining revealed that the viability of the islets stimulated with cytokines was significantly impaired as compared with that of the islets cultured without cytokines, whereas a marked improvement in viability was observed in the XBJ treatment group (**Figures 3E, F**). Similarly, after 24 hours of exposure to the inflammatory cytokine stimulus, XBJ significantly reduced the proportion of apoptotic islet cells, as quantitatively demonstrated by a decrease in TUNEL-positive cells from 25.38% in the cytokines group to 7.52% in the XBJ group (**Figures 3G, H**).

Moreover, the GSIS was conducted to evaluate the function of the islets. Results demonstrated that cytokine stimulation impaired insulin secretion, while XBJ restored the glucose responsiveness of islets, as indicated by an improved stimulation index (**Figure 3I**). After the damage of cytokines, the mouse islets have lost the ability to release insulin stimulated by high level glucose, and islets also have uncontrolled insulin release incubating in lower glucose. Conversely, XBJ reversed this phenomenon through ameliorating inflammatory damage (**Figure 3J**). Collectively, the results provide evidence that the application of XBJ considerably mitigates injury *in vitro* under cytokines stimulation.

### XBJ suppresses NF-κB pathway to attenuate cytokine-induced apoptosis based on RNA-Seq analysis

**Figure 4:**
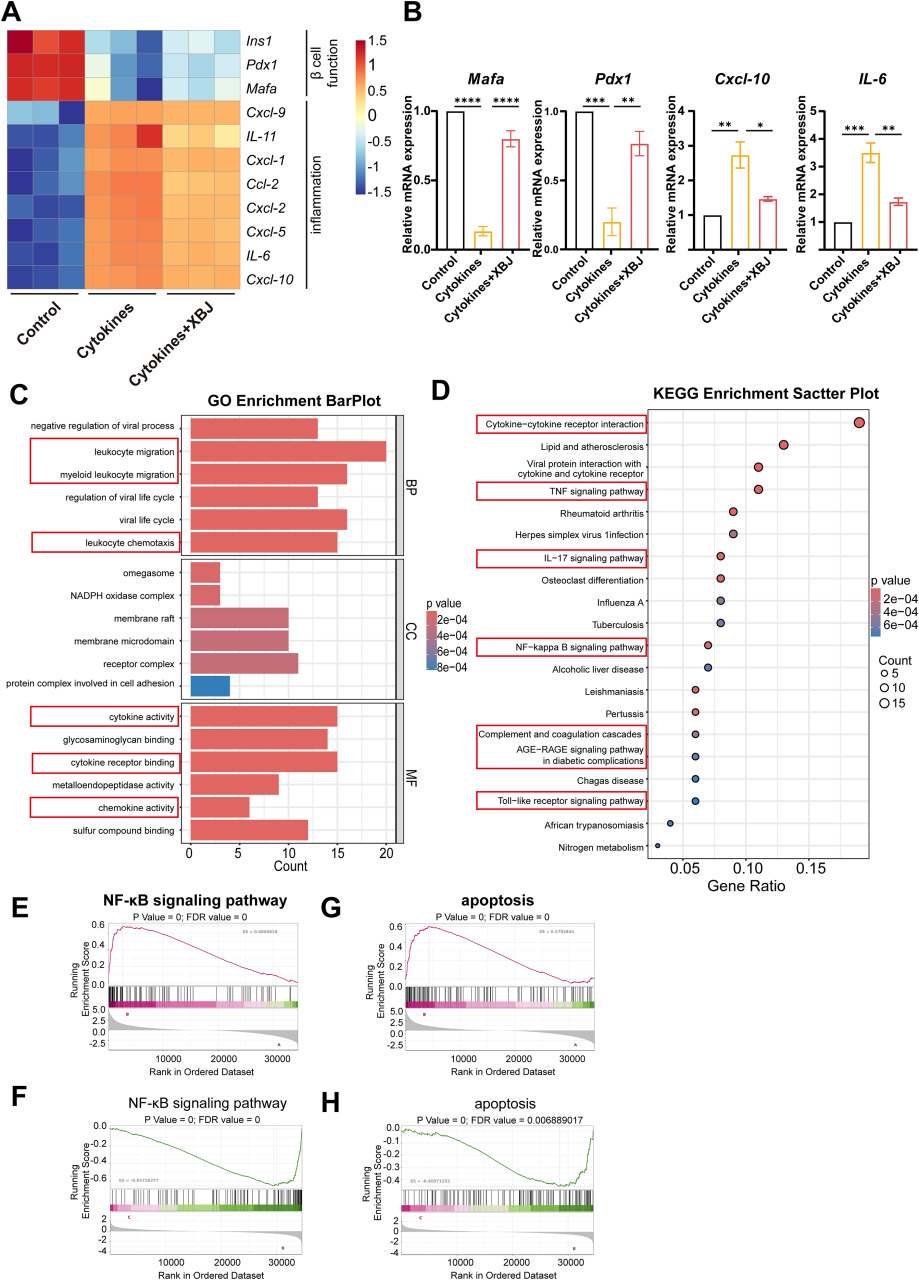
RNA-seq analysis of mouse islets under cytokines stimulation with or without XBJ. **(A)** The heatmap displays the response to inflammation and function-related genes in the control, cytokines and cytokines+XBJ groups. **(B)** qRT-PCR analysis of mouse islets under cytokines stimulation *in vitro* (n=3). GO analysis **(C)** and KEGG analysis **(D)** of change genes among control, cytokines and cytokines+XBJ groups. GSEA analysis of NF-κB signaling pathway. **(E, F)** and apoptosis **(G, H)** genes enriched in KEGG from RNA sequence among three groups. . *p <0.05, **p<0.01, ***p<0.001, ****p<0.0001.

To further explore the mechanism of the protective effects of XBJ on islets under cytokines stimulation, we performed RNA-sequencing (RNA-seq) analysis. Principal component analysis (PCA) demonstrated distinct clustering of the three groups in separate quadrants, indicating divergence in their global transcriptional profiles under differential treatment conditions (**Supplementary Figure S2A**). The volcano plot illustrated the significant differential expressed genes in transcript levels of cytokines group vs cytokines+XBJ group were analyzed (**Supplementary Figure S2B**). Genes of representative were screened from the differentially expressed genes, and the heatmap showed that classical β cell function genes were downregulated under cytokines stimulation, while treatment with XBJ protect β cell function (**Figure 4A**). XBJ also inhibited the expression of pro-inflammatory cytokine genes caused by cytokines (**Figure 4A**). These RNA-seq results were subsequently confirmed via qRT-PCR experiments (**Figure 4B**).

Further analysis identified 222 differentially expressed genes (DEGs) that either exhibited upregulation under cytokines stimulation followed by XBJ-induced downregulation (20 genes), or demonstrated suppression cytokines stimulation with subsequent reversal through XBJ treatment (202 genes). As shown in **Figure 4C**, the functional enrichment analysis showed that the DEGs were significantly enriched in leukocyte migration, myeloid leukocyte migration, leukocyte chemotaxis, cytokine activity, cytokine receptor binding, chemokines activity in GO pathways, which play crucial roles in IBMIR reaction (**Figure 4C**). Enrichment analysis in KEGG pathways demonstrated that enrichment of these genes in multiple inflammatory signaling pathways, with NF-κB signaling pathway representing the most prominent node, alongside coordinated activation of Toll-like receptor and TNF signaling pathways (**Figure 4D**). Furthermore, gene set enrichment analysis (GSEA) of KEGG pathways confirmed that XBJ inhibited inflammatory cascades triggered by each cytokine utilized in our inflammatory model (**Supplementary Figure S2C-H**). GSEA also demonstrated that XBJ concurrently suppressed both NF-κB pathway activation and cytokine-induced apoptosis (**Figures 4E–H**), suggesting a potential mechanism basis for its therapeutic efficacy in ameliorating outcomes of intrahepatic islet transplantation.

### XBJ inhibits inflammation-induced NF-κB activation and apoptosis

**Figure 5:**
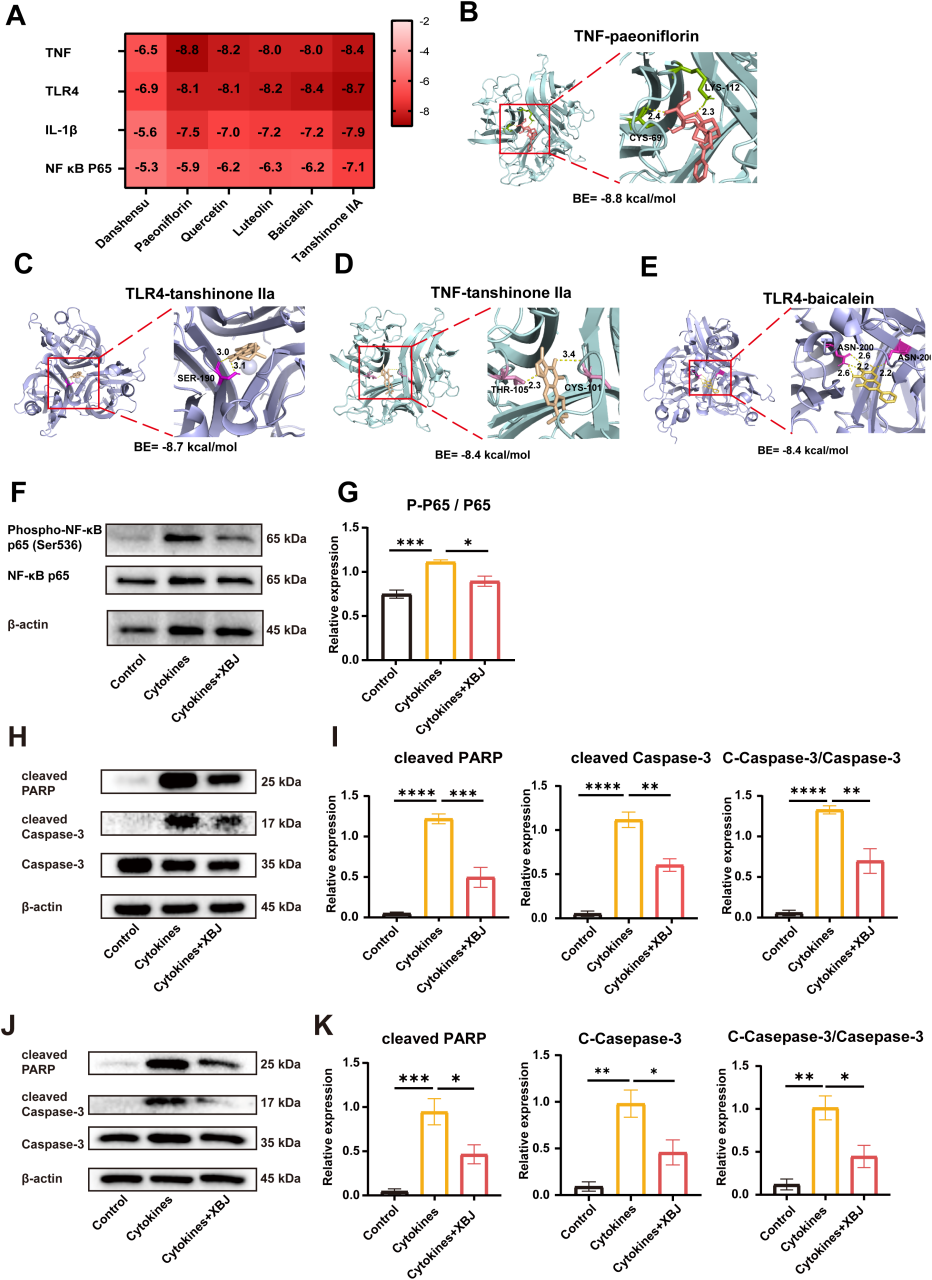
XBJ alleviates apoptosis under cytokines stimulation through NF-κB signaling pathway. **(A)** The heatmap of docking scores of NF-κB signaling pathway key targets combining with 6 active compounds. **(B-E)** Representative docking complexes of key targets with compounds. **(F, G)** Western blot analysis of phospho-NF-κB P65 and P65 of NIT-1 cells *in vitro* model (n=3). **(H-I)** Western blot analysis of cleaved PARP, cleaved Caspase-3 and Caspase-3 of NIT-1 cells *in vitro* model (n=4). **(J, K)** Western blot analysis of cleaved PARP, cleaved Caspase-3 and Caspase-3 of mouse islets *in vitro* model (n=4). *p<0.05, **p<0.01, ***p<0.001, ****p<0.0001.

Based on the results of RNA-seq analysis, suppression of NF-κB signaling pathway played a pivotal role in mediating XBJ’s protective effects. Molecular docking simulations predicted high-affinity interactions between anti-inflammatory phytochemicals in XBJ (e.g., danshensu, paeoniflorin, quercetin, luteolin, baicalein and tanshinone IIa) and protein products of both differentially expressed genes in RNA-seq and core NF-κB pathway components, suggesting a multi-target pharmacological mechanism. The results demonstrated successful docking in all 24 ligand-receptor pairs, with binding energies consistently below -5 kcal/mol (range: -5.23 to -8.8 kcal/mol), suggesting that bioactive constituents in XBJ may inhibit NF-κB pathway activation through multi-target engagement with key nodal proteins (**Figure 5A**). The top four ligand-receptor pairs with substantial binding affinities were identified as TNF-paeoniflorin, TLR4-tanshinone IIA, TNF-tanshinone IIA, and TLR4-baicalein, all exhibiting binding energies below -8 kcal/mol (**Figures 5B–E**).

To validate XBJ-mediated suppression of NF-κB signaling, Western blot analysis was applied in further studies. This result revealed that XBJ significantly reduced cytokine-induced phosphorylation of p65 at Ser536, demonstrating potent suppression of NF-κB pathway activation (**Figures 5F, G**).

Apoptosis assay detecting cleaved-PARP and cleaved-Caspase-3 by Western Blot were performed, and the results were consistent with the cell viability and NF-κB signal pathway statement. The expression of apoptosis markers was significantly higher in NIT-1 cells treated with cytokines compared to the control group, but a significantly lower rate was observed in the XBJ treatment group (**Figures 5H, I**).

This protective effect was further validated in mouse islets. After 24 h of cytokines stimulation, the expression of cleaved-PARP and cleaved-Caspase 3 was significantly increased in cytokines group. Conversely, in the XBJ treatment group, the opposite trend was observed (**Figures 5J, K**).

### XBJ enhances islet grafts survival and function in diabetic mice

**Figure 6:**
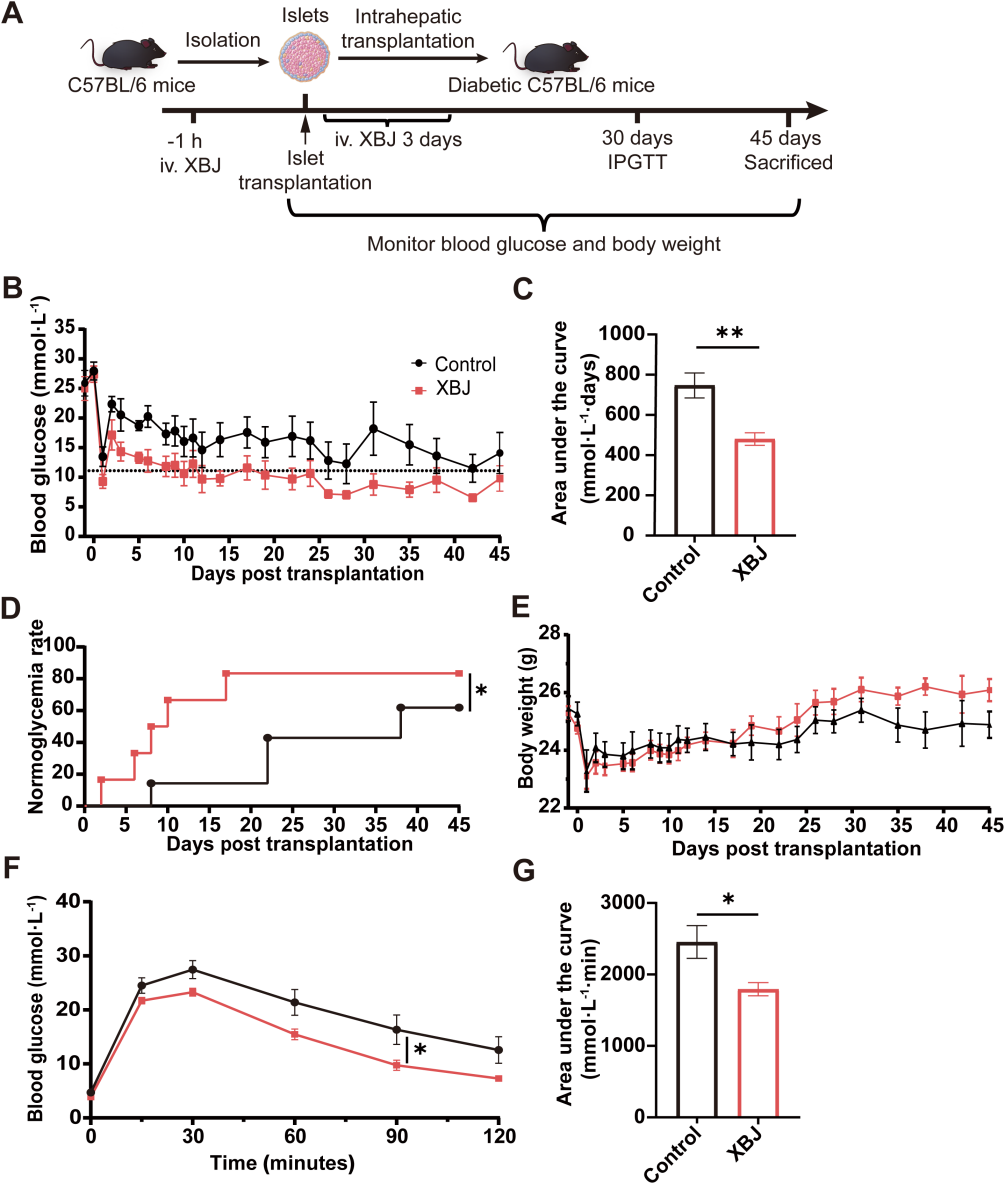
XBJ treatment improves islet grafts survival and function in intrahepatic islet transplantation model. **(A)** Schematic representation of the syngeneic islet transplantation timeline. **(B)** Non-fasting blood glucose levels after islet transplantation in diabetic recipient mice (n=6-7). **(C)** Area under curve (AUC) calculated based on the blood glucose levels. **(D)** Cure rate after islet transplantation in recipient mice. **(E)** Body weight after islet transplantation in diabetic recipient mice (n=6-7). **(F)** Intraperitoneal glucose tolerance test (IPGTT) at 30 days post-transplantation. **(G)** AUC of the IPGTT curve. Data are presented as the mean ± SEM, n=6–7 for each group. *p<0.05, **p<0.01.

To explore the effect of XBJ on improving the outcome of islet transplantation, we established a syngeneic intrahepatic islet transplantation model (**Figure 6A**). When we transplanted the marginal dose syngeneic C57BL/6 mice islets into diabetic mice, only 57.1% of recipients achieved normoglycemia at 45 days after transplantation in the control group. In contrast, in the group treated with XBJ, the cure rate significantly increased to 85.7% at the same time point (**Figures 6B–D**). Moreover, the blood glucose of cured mice treated with XBJ decreased to normal levels more rapidly than that in the control group after transplantation (**Figure 6D**), and the blood glucose value of XBJ treatment group was significantly lower than the control group (**Figure 6C**). After transplantation, the body weight of all mice showed an upward trend, and this trend was more pronounced in the XBJ treatment group (**Figure 6E**).

To assess the function of the graft’s response to blood glucose in each group, all recipients received intraperitoneal glucose tolerance tests (IPGTT). Compared with control group, the mice in XBJ treatment group exhibited superior glucose tolerance, returning to baseline glucose levels within 120 minutes post-injection (**Figure 6F**). Additionally, the area under curve (AUC) for XBJ treatment group was significantly less than that of control groups (**Figure 6G**), further supporting improved graft function.

**Figure 7:**
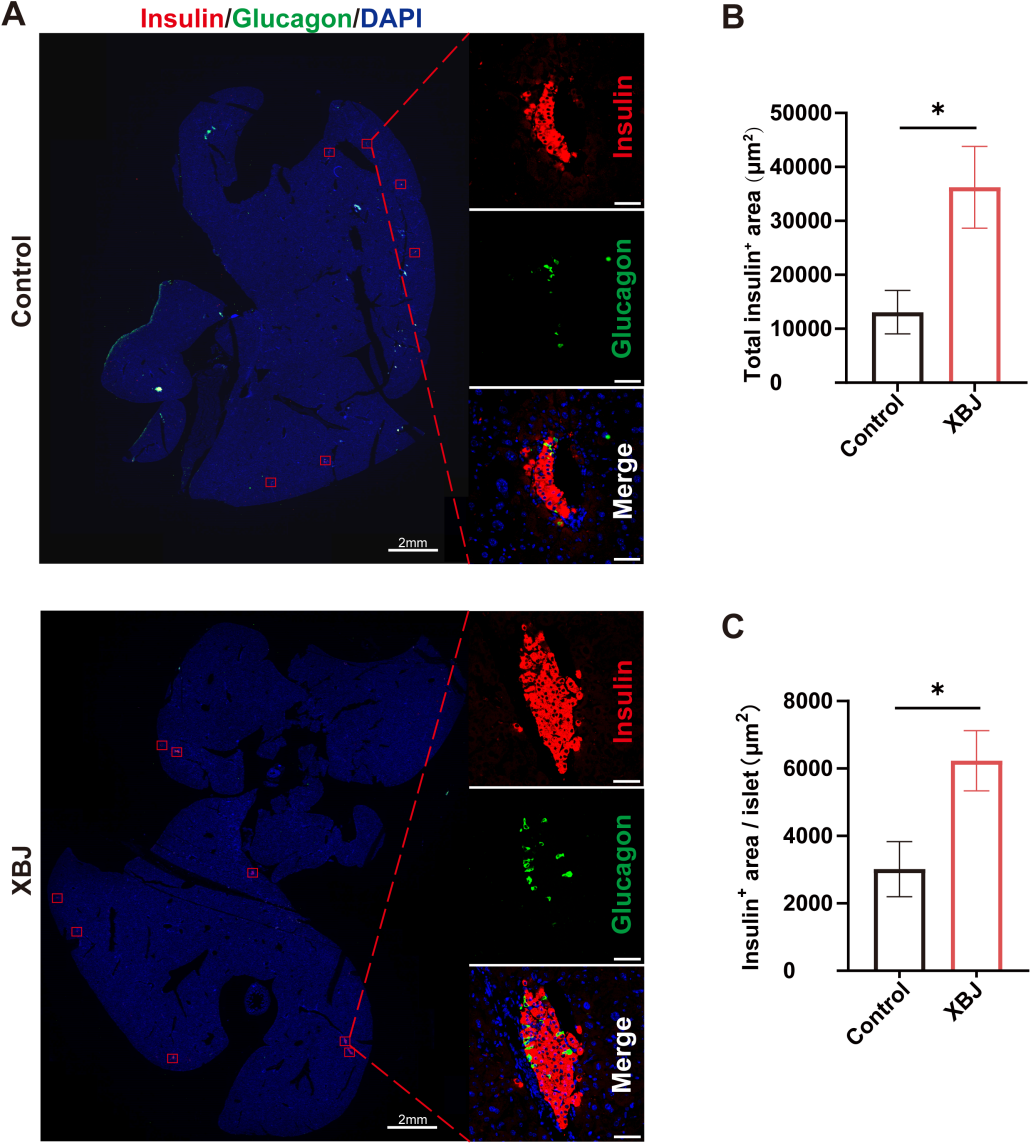
Recipients receiving XBJ treatment had larger islet mass at 45 days post-transplantation. **(A)** Representative images of immunofluorescence staining of islet grafts at 45 days post-transplantation. **(B, C)** Quantitative analysis of total insulin positive area for each slide **(B)** and insulin positive area for each islet **(C)**. Data are presented as the mean ± SEM, from 6–7 recipients each group. Scale bar, 50μm. *p<0.05.

All mice were sacrificed for histological assessment of islet survival and mass in the liver at 45 days post-transplantation. We performed random immunofluorescence staining of the whole liver section with anti-insulin and anti-glucagon antibodies for each mouse with islet grafts (**Figure 7A**). Quantification revealed a significantly larger total insulin-positive area in the XBJ treatment group compared to the control group, indicating improved islet engraftment and survival (**Figure 7B**). Moreover, individual islet area was significantly larger in the XBJ-treated group compared to controls, suggesting better preservation of islet structure (**Figure 7C**). These results demonstrated that XBJ treatment significantly improved the function and maintain the structural integrity of islet grafts in intrahepatic transplantation.

## Discussion

This study demonstrates that XBJ can mitigate IBMIR and enhances intrahepatic islet transplantation. XBJ not only mitigates the detrimental impact of IBMIR but also directly preserves islet cell viability and function. *In vivo*, XBJ reduced peri-islet thrombosis, immune cell infiltration and apoptosis, while preserving islet integrity and function. In parallel, *in vitro* studies using NIT-1 cells and primary mouse islets revealed that XBJ directly counteracts cytokine-induced apoptosis and dysfunction. Mechanistically, RNA sequencing and molecular docking revealed high-affinity binding of XBJ constituents to NF-κB pathway nodes. Collectively, these findings establish XBJ as a novel, multi-target therapeutic candidate that addresses both inhibition of IBMIR and directly protection of islets during transplantation.

Intrahepatic islet transplantation via the portal vein is the most commonly employed method for clinical islet transplantation (23). However, the islet grafts are immediately exposed to the recipient’s blood upon infusion into the liver using this method (24). This direct contact with blood triggers the IBMIR, a critical barrier to successful islet transplantation (5, 25). We demonstrate for the first time that herbal injection suppresses IBMIR and directly protects islets in the intrahepatic islet transplantation model. Current therapeutic approaches and preclinical studies, primarily involving TNF-α blocker (e.g., Etanercept), IL-1β receptor antagonists and α1-antitrypsin inhibitors, have been clinically adopted by many transplant centers (26–31). However, despite these advances, clinical efficacy remains limited (27, 32), primarily due to the cascade amplification effect of IBMIR, which involves rapid and overlapping activation of inflammation, coagulation, and complement pathways (33). Inhibiting a single factor within this complex network may not be sufficient to achieve significant clinical improvement, as blocking one pathway does not prevent the activation and amplification of others (34).

In contrast, the multi-target and multi-component nature of traditional Chinese medicine compounds, such as XBJ, offers a promising alternative to address the multifaceted nature of IBMIR. XBJ contains multiple active constituents such as danshensu, paeoniflorin, baicalein, luteolin, quercetin, and tanshinone IIA, which have been reported to inhibit inflammatory signaling. Specifically, these compounds suppress the NF-κB (35) and KEAP1/NRF2 (36) pathways, and modulate apoptosis- and cytokine-related targets including BCL2, IL-6, CXCL8, and IL-1β (37–40). These reported mechanisms are consistent with our molecular docking and RNA-seq findings (**Figure 5**), supporting that XBJ alleviates IBMIR through multi-target inhibition of NF-κB driven inflammatory cascades. Furthermore, as a standardized injection, XBJ is formulated for intravenous delivery to ensure rapid and complete systemic bioavailability of its active compounds, which is essential for achieving timely therapeutic concentrations in the liver, the site of islet engraftment. This pharmacokinetic advantage is critical for intervening in IBMIR. This unique feature positions XBJ as a potentially more effective approach to inhibiting IBMIR and preserving islet viability. Our study demonstrates that XBJ, through its ability to suppress key inflammatory response and prevent islet from death stimulated by cytokines stimulate, could offer a novel solution to this challenge, preserving islet viability and enhancing graft function in the immediate post-transplant period.

The pathogenesis of IBMIR is primarily driven by the rapid activation of innate immune responses and coagulation pathways upon islet exposure to blood (6, 41). These two processes act synergistically, forming a cascade amplification loop that accelerates islet destruction (25). Our study demonstrates that XBJ effectively disrupts this pathological interplay by simultaneously suppressing inflammatory cell infiltration and reducing thrombus formation around transplanted islets. Histological analyses revealed a marked decrease in leukocyte accumulation and peri-islet thrombosis following XBJ treatment, indicating dual inhibition of key IBMIR pathways. This coordinated anti-inflammatory and anti-coagulant action alleviates early islet injury and contributes to improved graft preservation. The XBJ-mediated upregulation of Mafa/Pdx1 and islet mass expansion reflect functional preservation of existing β-cells rather than regeneration. This is supported by the low proliferative capacity of mature islets (42), the coordinated enhancement of functional markers with insulin secretion, and the correlation between graft size and IBMIR attenuation, collectively indicating that XBJ protects islets from inflammatory damage. In recent years, various strategies have been explored to protect islets from apoptotic destruction of IBMIR, such as over-expression of anti-apoptotic genes (e.g., A20 or Bcl-2), use of caspase inhibitors (e.g., ZVAD-FMK and IDN-6556), and treatment with glucagon-like peptide-1 (GLP-1) analogs (e.g., Exendin-4 and Liraglutide) (43–48). Our findings indicate that XBJ provides significant protection to islet cells. Under cytokine-induced stress, XBJ effectively reduced apoptosis in both NIT-1 cells and primary islets, as evidenced by the decreased the percentage of TUNEL positive cells and expression of apoptosis markers such as cleaved-caspase 3 and cleaved PARP. Furthermore, XBJ preserved islet functionality, maintaining insulin secretion in response to glucose stimulation, which underscores its ability to protect islet viability and function in an inflammatory environment.

Beyond its distinct anti-inflammatory and cytoprotective properties, the dual mechanisms of XBJ appear to act collaboratively to improve transplantation outcomes. The ability to concurrently suppress IBMIR-associated inflammatory damage and directly enhance islet resilience under cytokine stress may offer superior benefits compared to conventional single-target therapies. This integrative approach addresses both systemic and cellular-level threats encountered during early islet engraftment, highlighting the multifaceted potential of XBJ in clinical transplantation settings. The demonstrated benefits of XBJ are specific to the intraportal model, wherein it primarily counteracts IBMIR. We would therefore anticipate minimal efficacy in sites that avoid blood-mediated inflammation, such as the renal subcapsule, as the primary pathological target of XBJ is absent. This delineates XBJ’s potential application specifically for clinical intraportal transplantation, while its IBMIR-independent effects in extrahepatic sites warrant future investigation. Our findings highlight the clinical translational potential of XBJ as an adjunctive therapy to improve the efficacy of islet transplantation. While XBJ has been extensively used in clinical settings for treating systemic inflammatory conditions such as sepsis, its application in the field of islet transplantation has not yet been explored. This study is the first to provide comprehensive evidence that XBJ can target the key pathological mechanisms of IBMIR, suggesting its potential role in reducing early islet loss and enhancing graft function. In addition, we will further explore the potential of XBJ to improve the outcomes of stem cell-derived islet transplantation in future studies. Given that XBJ is already an approved and widely used clinical medication, it offers a practical and promising option for rapid translation into clinical protocols for islet transplantation, potentially accelerating progress toward improved clinical outcomes in islet transplantation.

## Data Availability

The data presented in this study are deposited in the SRA repository (BioProject: PRJNA1390313).
