# Supplementary material for: Xuebijing injection mitigates instant blood−mediated inflammatory reaction and enhances intrahepatic islet transplantation via target NF−κB pathway
Source: Front Immunol. 2025 Dec 23;16:1671966. doi: 10.3389/fimmu.2025.1671966 (PMC12771108; doi:10.3389/fimmu.2025.1671966)
Supplement: Supplementary file 1 [file DataSheet1.pdf]

Supplementary table 1. Primers for qRT-PCR

Primers for qRT-PCR		
**Genes**	**Forward Primers (5'-3')**	**Reverse Primers (5'-3')**
β-actin	GTGACGTTGACATCCGTAAAGA	GCCGGACTCATCGTACTCC
TNF-α	CCCTCACACTCAGATCATCTTCT	TGCTACGACGTGGGCTACAG
Ccl-2	CTGTCAATGCCTGAAGACC	GCAAACTTTTTGACCGCC
Mafa	AGGAGGAGGTCATCCGACTG	CTTCTCGCTCTCCAGAATGTG
Cxcl-10	CCAAGTGCTGCCGTCATTTTC	GGCTCGCAGGGATGATTTCAA
IL-6	AGCCAGAGTCCTTCAGA	GGTCCTTAGCCACTCCT
Pdx1	CCCCAGTTTACAAGCTCGCT	CTCGGTTCCATTCGGGAAAGG

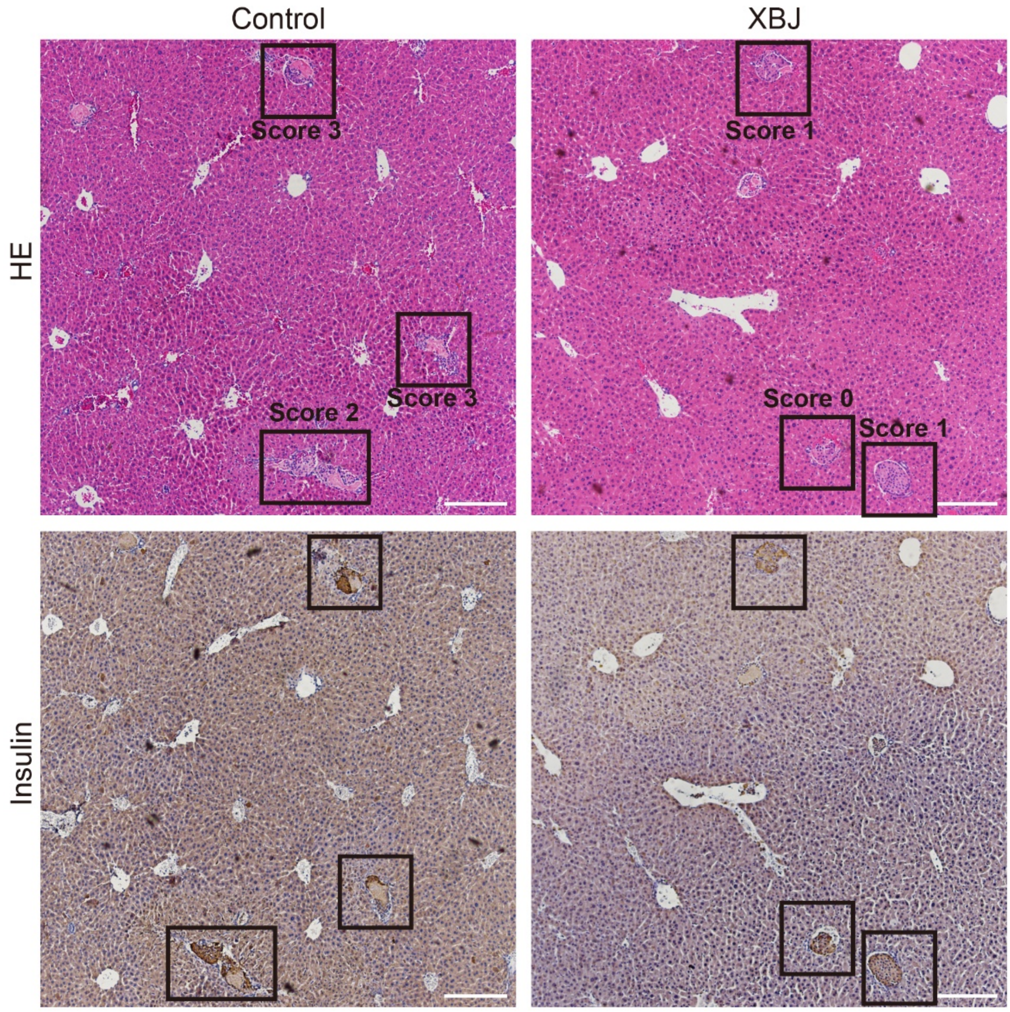

Supplementary Figure 1. The XBJ treatment group generally had a lower thrombus deposition score. Representative H&E staining (upper) and immunohistochemical staining images of insulin (lower) in liver sections after 6 hours post-transplantation from both groups.

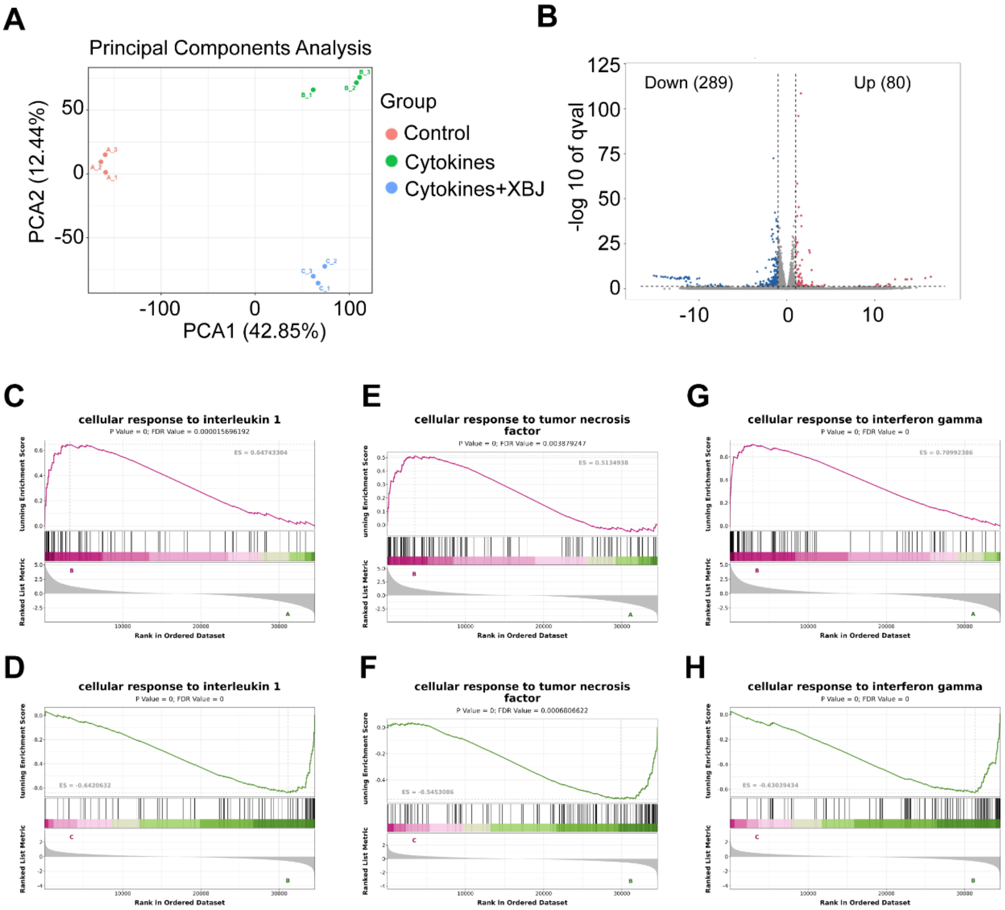

Supplementary Figure 2. Gene and signaling pathway analysis among control, cytokines and cytokines+XBJ groups in vitro. (A) PCA analysis of three groups. (B) Volcano plot of differentially expressed genes between cytokines and cytokines+XBJ group. (C)-(H) GSEA analysis of changed genes enriched in KEGG.
